# Three-Dimensional Imaging of a Central Venous Dialysis Catheter Related Infected Thrombus

**DOI:** 10.1155/2015/724132

**Published:** 2015-11-24

**Authors:** Diana Yuan Yng Chiu, Darren Green, Philip A. Kalra, Nik Abidin

**Affiliations:** ^1^Vascular Research Group, Institute of Population Health, The University of Manchester, Manchester Academic Health Sciences Centre, Manchester M13 9PL, UK; ^2^Department of Renal Medicine, Salford Royal NHS Foundation Trust, Stott Lane, Salford M6 8HD, UK; ^3^Department of Cardiology, Salford Royal Hospital, Salford Royal NHS Foundation Trust, Stott Lane, Salford M6 8HD, UK

## Abstract

Three-dimensional (3D) echocardiography is becoming widely available and with novel applications. We report an interesting case of a 68-year-old lady with a central venous thrombosis coincident with both a dialysis catheter infection and a recent pacemaker insertion. Two-dimensional transesophageal echocardiography was unable to delineate whether the thrombosis was involved with the pacemaker wire or due to the tunneled catheter infection. The use of 3D echocardiography was able to produce distinct images aiding diagnosis. This circumvented the need for invasive investigations and inappropriate, high-risk removal of the pacing wire. This case highlights the emerging application of 3D echocardiography in routine nephrology practice.

## 1. Introduction

Central venous catheters are a crucial form of access for hemodialysis, especially when providing acute dialysis therapy or as a therapeutic bridge whilst waiting for an arteriovenous graft formation. But dialysis catheters are not without risks. Thrombosis is one well-recognized complication, second in prevalence to catheter related infection. Contrast venography is considered the gold standard for visualization of central venous thrombosis. However, this is invasive and associated with contrast and radiation exposure. We present the case of a lady who had a dialysis catheter related thrombus in which two-dimensional (2D) imaging could not delineate the extent of thrombus and the novel application of three-dimensional (3D) echocardiography aided diagnosis.

## 2. Case Presentation

### 2.1. Clinical History and Initial Laboratory Data

A 68-year-old lady with End Stage Kidney Disease (ESKD) secondary to IgA nephropathy had been established on maintenance hemodialysis for three years via a tunneled central venous dialysis catheter. She presented with fever after a routine dialysis session having had a change of tunneled dialysis catheter due to a split in the catheter 6 weeks before. She had no focal symptoms such as dysuria or productive cough to indicate an alternative source of infection. Other medical history included type 2 diabetes mellitus, hypothyroidism, and an aortic tissue valve replacement for severe aortic stenosis two months previously. This was complicated by complete heart block and a pacemaker inserted. She was a nonsmoker and did not drink alcohol. Medications were simvastatin and levothyroxine. On physical examination, the patient was febrile with temperature 38.2°C, blood pressure 112/72 mmHg, heart rate 105 beats/min, respiratory rate 20 breaths/min, and oxygen saturations 99% on air. Her chest was clear on auscultation, jugular venous pressure was not raised, and cardiac heart sounds were heard with a grade 2 apical pansystolic murmur. She had pitting sacral edema, but no splinter hemorrhages, Roth's spots, or Janeway lesions.

Laboratory tests showed albumin 3 g/dL, hemoglobin 8.4 g/dL, white blood cell count 3.7 × 10^3^/*μ*L, platelets 86 × 10^3^/*μ*L, and C-reactive protein 126 mg/L. Urine culture displayed mixed growth without pyuria. An electrocardiogram showed sinus rhythm, and a plain chest radiograph confirmed a right tunnelled central venous dialysis catheter and left pacemaker wire* in situ*, but no other abnormalities. Central and peripheral blood cultures were positive with* Staphylococcus aureus* sensitive to vancomycin, gentamicin, and rifampicin.

### 2.2. Imaging Studies

**Figure 1:**
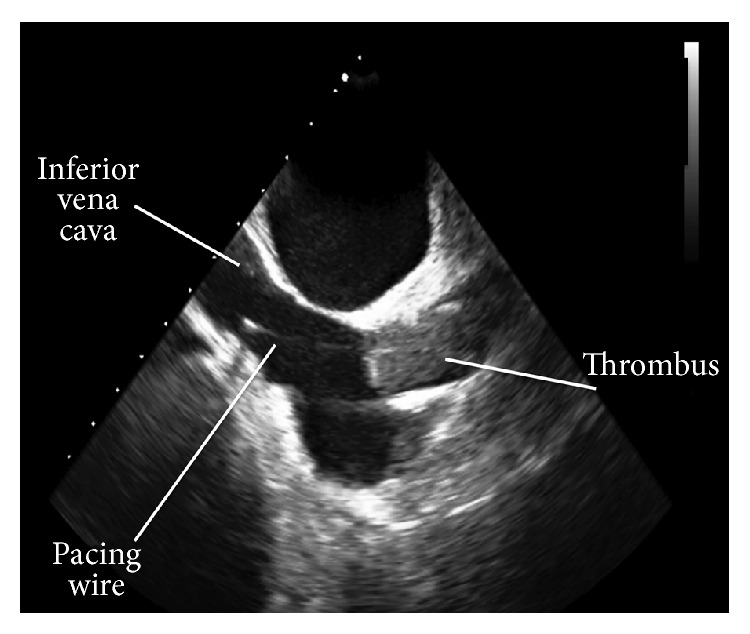
Transesophageal echocardiogram demonstrating a large thrombus in the superior vena cava. The pacing wire appears to overlay the thrombus. Because this is a 2D image, hence no spatial depth, it is unclear if there is a space between the thrombus and wire or whether it is independent of this.

**Figure 2:**
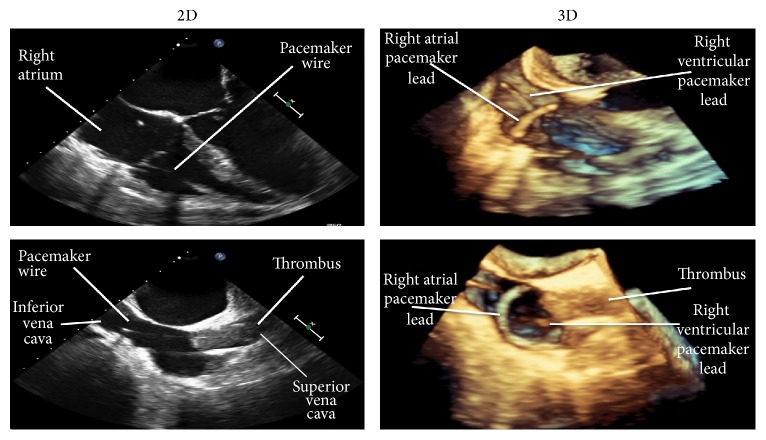
3D echocardiogram demonstrated the tract of the pacemaker wires clearly and showed that the venous thrombosis was separate from the pacemaker wires. In comparison, 2D echocardiography is less distinct.

Dialysis catheter associated infection with possible infective endocarditis was the presumed diagnosis and therefore the central venous dialysis catheter was removed under local anesthetic. The patient then underwent a 2D transesophageal echocardiogram that demonstrated normal heart valves but showed a large thrombus at the superior vena cava/right atrial junction with some protrusion into the right atrial cavity and in proximity to her pacing wire. It was not possible to delineate the spatial relationship of the thrombus to the pacemaker wire because the ultrasound image can only depict a sector, originating from the rotation axis of the transducer (Figure 1). It was therefore not clear whether the infection and/or thrombus was caused by or involved the pacing wire. This is a vital consideration as there would be increased technical difficulty and risk of thrombus dislodgement for pacemaker removal if the thrombus was entangled with the pacemaker wire. Therefore, using the same echocardiography machine and with selection of the 3D function (echocardiography machine, Philips IE 33 x MATRIX; Transducer: X5-1; 3D analysis software: QLAB 9), 3D images were acquired (Figure 2). These images clearly demonstrated the tracts of the pacemaker wires (2 pacing leads: right atrial and right ventricular leads) and showed that the venous thrombosis was separate from the wires.

### 2.3. Diagnosis

A diagnosis of infected venous thrombosis without involvement of pacemaker wire was therefore reliably made. This meant that there was no need for further invasive investigation and also that no inappropriate, high-risk attempts to remove and replace the pacing wire were undertaken.

### 2.4. Clinical Follow-Up

The patient received sensitivity-specific antibiotics for 4 weeks and was commenced on peritoneal dialysis. She was warfarinized to an international normalized ratio target of 2.5 intended for lifelong treatment. Her fever settled and she was discharged without the need for pacemaker removal.

## 3. Discussion

The reported incidence of central catheter venous thrombosis varies between 1.5 and 33% depending on the definition [1, 2]. Factors that predispose to thrombosis are described in Virchow's triad: hypercoagulability, hemodynamic disturbance (stasis or turbulence), and endothelial damage. The risk factors associated with tunneled dialysis catheters are multiple lumen [3], catheters* in situ* for >2 weeks, and multiple previous catheter insertions at the same site. Our patient had a recent pacemaker insertion, cardiac surgery, and exchange of tunneled dialysis catheter and therefore had repeated vascular trauma.

In this case, the thrombosis was further complicated by infection. The fibrin sheath formed around the catheter lumen and external portions provide an ideal surface for microorganisms such as* Staphylococcus aureus* and* Staphylococcus epidermidis* to adhere to [4]. In turn, the infection increases the incidence of thrombosis.

The novelty in this case rests on the use of 3D echocardiographic imaging to delineate the course of the thrombosis. Contrast venography is considered the gold standard for diagnosis of catheter related deep vein thrombosis. However, this investigation requires radiation exposure and intravenous contrast which may be problematic for dialysis patients who are sensitive to a fluid load or allergic to contrast agents. Furthermore, the image obtained with venography may only demonstrate a filling defect and it may be difficult to visualize the exact involvement of wires in relation to the thrombus.

For this patient the thrombosis was identified by transesophageal echocardiography performed to detect infective endocarditis. This was easy to perform and did not require intravenous contrast. However, 2D transesophageal echocardiography could not accurately delineate the course of the thrombosis (Figure 1). Due to inadequate spatial visualization it is unclear whether the pacemaker wire is entangled with the thrombus or independent of this, but with 3D echocardiography a clear diagnosis was obtained. Moreover, 3D echocardiography was carried out in the same sitting with the same machine.

Real time 3D echocardiography is an advanced technique that is becoming widely available in clinical practice. A 3D image is acquired in a manner similar to 2D echocardiography with a specialised transducer. A pyramidal volume of tissue is scanned with the apex at the probe. The images may then be manipulated in different ways by the echocardiographic software to allow visualization from different angles. Image may be rotated in three different axes (viewed from top to bottom, front to back, or left to right) or progressively sliced to the plane of interest. As a result, the depth of the image may be appreciated much better than 2D echocardiographic images and provides better visualization of adjacent cardiac structures and masses (e.g., thrombus). For this patient, use of 3D imaging subverted the need for the invasive contrast venography.

The image produced by 3D is easy to interpret, even by a nonspecialist (Figure 2). A more common application for 3D echocardiography is in measurements of left ventricular mass and function. Limitations of 2D echocardiography in determining left ventricular ejection fraction and mass are that it involves calculations that assume the left ventricle to be elliptical in shape and frequently 2D echocardiography Forshortens the left ventricular apex; hence, there will be inaccuracies in the measurements. 3D echocardiography does not make any geometric assumptions and hence has been shown to produce calculations of left ventricular volume and ejection fraction comparable to the gold standard of cardiac magnetic resonance [5, 6]. Furthermore, there is reduced interobserver variability and high reproducibility compared with conventional 2D echocardiography [7]. As a result, this has been utilized in research studies in patients with chronic kidney disease and end stage renal failure for more accurate cardiac volume assessments [8, 9]. In this case, we demonstrate another useful application of 3D echocardiography in patients with ESKD undergoing hemodialysis via a tunneled venous catheter.

Treatment of tunneled dialysis catheter related thrombosis prevents later complications such as pulmonary embolism, vascular compromise of the limb, or death. There is no standardized method of treatment, although it may involve thrombolysis, systemic anticoagulants, and/or removal of catheter [10]. In this case, the lady responded well to treatment, dialysis catheter was removed safely, and the pacing wire was left* in situ* with no adverse events.

In conclusion, we present a case whereby the use of 3D echocardiography has provided an accurate diagnosis and aided management for an infected dialysis catheter related venous thrombosis. It is likely that further clinical applications may be further defined for this useful investigation.
